# State-of-Art Bio-Assay Systems and Electrochemical Approaches for Nanotoxicity Assessment

**DOI:** 10.3389/fbioe.2020.00325

**Published:** 2020-04-28

**Authors:** Ravikumar B. Shinde, Murugan Veerapandian, Ajeet Kaushik, Pandiaraj Manickam

**Affiliations:** ^1^Department of Biotechnology, Rajarshi Shahu College, Latur, India; ^2^Electrodics and Electrocatalysis Division, CSIR-Central Electrochemical Research Institute (CECRI), Academy of Scientific and Innovative Research (AcSIR), Ghaziabad, India; ^3^NanoBioTech Laboratory, Department of Natural Sciences, Division of Sciences, Art & Mathematics, Florida Polytechnic University, Lakeland, FL, United States

**Keywords:** nanomaterials, toxicological effects on human, biological assay models, molecular mechanism of toxicity, bioelectrochemical methods

## Abstract

Innovations in the field of nanotechnology, material science and engineering has rendered fruitful utilities in energy, environment and healthcare. Particularly, emergence of surface engineered nanomaterials offered novel varieties in the daily consumables and healthcare products including therapeutics and diagnostics. However, the nanotoxicity and bioactivity of the nanomaterials upon interaction with biological system has raised critical concerns to individual as well as to the environment. Several biological models including plant and animal sources have been identified to study the toxicity of novel nanomaterials, correlating the physio-chemical properties. Biological interaction of nanomaterials and its mediated physiological functions are studied using conventional cell/molecular biological assays to understand the expression levels of genetic information specific to intra/extra cellular enzymes, cell viability, proliferation and function. However, modern research still demands advanced bioassay methods to screen the acute and chronic effects of nanomaterials at the real-time. In this regard, bioelectrochemical techniques, with the recent advancements in the microelectronics, proved to be capable of providing non-invasive measurement of the nanotoxicity effects (*in vivo* and *in vitro*) both at single cellular and multicellular levels. This review attempted to provide a detailed information on the recent advancements made in development of bioassay models and systems for assessing the nanotoxicology. With a short background information on engineered nanomaterials and physiochemical properties specific to consumer application, present review highlights the multiple bioassay models evolved for toxicological studies. Emphasize on multiple mechanisms involved in the cell toxicity and electrochemical probing of the biological interactions, revealing the cytotoxicity were also provided. Limitations in the existing electrochemical techniques and opportunities for the future research focusing the advancement in single molecular and whole cell bioassay has been discussed.

## Introduction

Emergence of interdisciplinary research in materials science has revolutionized the advent of nanomaterials. Material with at least one dimension below 100 nm have got tremendous attention from researchers all over the world, due to their unique physicochemical properties. Nanomaterials with spherical, rod, hollow spheres, fibrous structures and sheets are the widely explored architectures for multi-functional applications. Customized nanoparticles with multi-layered structures comprising a central portion known as core, middle one or interface layer of chemically different from core material and the surface layer that can be functionalized by surfactants, small molecules, metal ions or different polymers are having wide varieties of applications in the emerging research area (Shin et al., 2016).

Nanomaterials are broadly categorized into carbonaceous, inorganic (metal/metalloid) and polymer classes. Owing to their abundant cost-efficient precursor source and scalable preparation methods, carbon-based nanomaterials have huge interest among materialist for fundamental and applied research. Particularly with recent studies on layered nanostructures of carbon had opened new avenues for two-dimensional derivatives such as graphene-like materials and metal chalcogenides. Similarly, metal and metalloid NPs were demonstrated for cancer therapeutics, diagnosis and antimicrobial applications (Sau and Rogach, 2010; Shakibaie et al., 2010; Sekhon, 2013). Particularly, superparamagnetic iron oxide and silver-silica composite NPs has widespread advantages for drug delivery, wound healing and anti-microbial applications (Veerapandian and Yun, 2013; Laurent et al., 2014). In this trend, integration of polymer species within the metal or metal oxide surfaces played critical role in enhancing the thermal stability, film forming capacity and hydrophobicity/hydrophilicity (Parveen and Sahoo, 2008; Karlsson et al., 2018). For instance, synthetic, semi-synthetic and natural material-derived polymers were explored in surface functionalization of metal/metal oxide NPs (Veerapandian and Yun, 2010; Safari and Zarnegar, 2014; Manickam et al., 2020). Such combination of two or three different chemical composition into single nanostructures are classified as hybrid NP system. Unlike single formulation, hybrid NPs enable synergistic properties of two or more individual materials. This includes, dual functionality like theranostics, i.e., therapy and diagnosis for fundamental research in cell biology, pathophysiology, drug discovery and disease diagnosis (Sailor and Park, 2012; Marimuthu et al., 2018). Further, scalability issues like production cost, shelf life and final property of the nanosystem can be amplified by hybridization or additive manufacturing technology (He et al., 2015). Solubility, reactivity, hardness, thermal stability, crystallinity and other physico-chemical/biological properties of NPs are indeed attributed to their size, shape and structural compositions. High surface-to-volume ratio of NPs offers huge advantages over the conventional bulk material hence it has wide applications in pharmaceutical and biomedical sectors (Wang et al., 2016), chemical/textile/food processing industries (Stark et al., 2015), energy harvesting/storage (Cassee et al., 2011) and other consumer products (Stamm et al., 2012). At this juncture, it is essential to be alert of consequences of new developments in this field. Hence, a better understanding of risks associated with the use of NPs especially characteristics of nanomaterials that are hazardous to human or environmental health must be identified.

Regardless of fundamental physical, chemical and biological characterization of specific NPs available in the literature still thorough toxicological studies are lack in different environment. For instance, the cytotoxicity effects (*in vitro*) of different forms of carbon nanomaterials has been reviewed (Yuan et al., 2019). Purification involving metals and acid functionalization of carbon based nanomaterials showed to increase their toxicity levels (McShan and Yu, 2014; Jang and Hwang, 2018). The major route of cytotoxicity induced by carbon NPs are through inhalation (Morimoto et al., 2012). The mode of synthesis, physical and chemical properties of carbon nanomaterials have significant impact on biological activity. Fiber length, surface area, particle size, agglomeration, and impurities present in the carbon nanomaterials during purification process (vide supra) also have influences on the biological activity and cytotoxicity effect of the carbon nanomaterials (Morimoto et al., 2012). On the other hand, oxidative stress and alteration of iron homeostasis is the major toxicity reported from superparamagnetic iron oxide NPs. Antibacterial agents like silver and its composite can lead to intracellular stress in lung, liver and kidneys. In some instance, cerium oxide-based composite has also enabled oxidative stress with respect to size (Khanna et al., 2015). The underlying limitation exists among the researchers in broad perspective is lack of modern analytical techniques for toxicological assay and convenient model system for exploration. Moreover, the reluctance between the different fields of expertise often hamper the interdisciplinary research which ultimately limits the feasibility or sustainability of novel materials.

Amongst variety of analytical techniques, in the recent past, electrochemical based nanomaterial characterization and bioassay has been unprecedentedly documented in the literature. Owing to the fact of simple instrumentation, handling of small sample volume, user-friendliness and point-of-care feasibility, electrochemical based analytical devices are widely explored. However, utilization of electrochemical approach in cell-cell interaction, celldrug interaction, cell cytotoxicity assessment, real-time analysis on animal models are at infant stage. The sensitivity and data interpretation pertaining to real samples with complex extracellular or intracellular matrix are challenging. Electrochemical methods have emerged as an important analytical tool for probing the biochemical events occurs in the cellular environment. It provides thermodynamic and kinetic parameters of cells under variation conditions. There are three important types of electrochemical analysis such as amperometric, potentiometric and impedimetric were widely used for cell biology studies. As depicted by their name, amperometric detectors measures the electrochemical activity of cells by monitoring the changes in current response, where are potentiometric sensors can probe the electrochemical activity in the cellular medium by measuring the electrode potential. Electrochemical impedimetric sensors measures the impedance of the cell medium as well as the impedance of the electrode. Different type of electrochemical methods has been employed to probe the various cellular cytotoxicity events. For example, amperometry and voltammetric techniques (sub classes of amperometric method) can be used to detect the redox active biochemical molecules (neurotransmitters, biogenic amines) released from cells. Amperometric measurements involves the biasing of working electrodes to oxidize or reduce the molecules in the solution. Amperometry can probe the change in concentration of small molecules oxidized or reduced at the electrode through current vs. time graph. Thus, the present review aimed to provide a frontier survey on state-of-art assay models and electrochemical techniques devoted in nanotoxicity assessment. Outcome of this report would not only benefit the material chemists' and environmental scientists' but also provide valuable resource to researchers working on bioassay platform design, biomaterials, pharmaceuticals and biomedical science.

### Biomedical Applications of NPs

**Figure 1:**
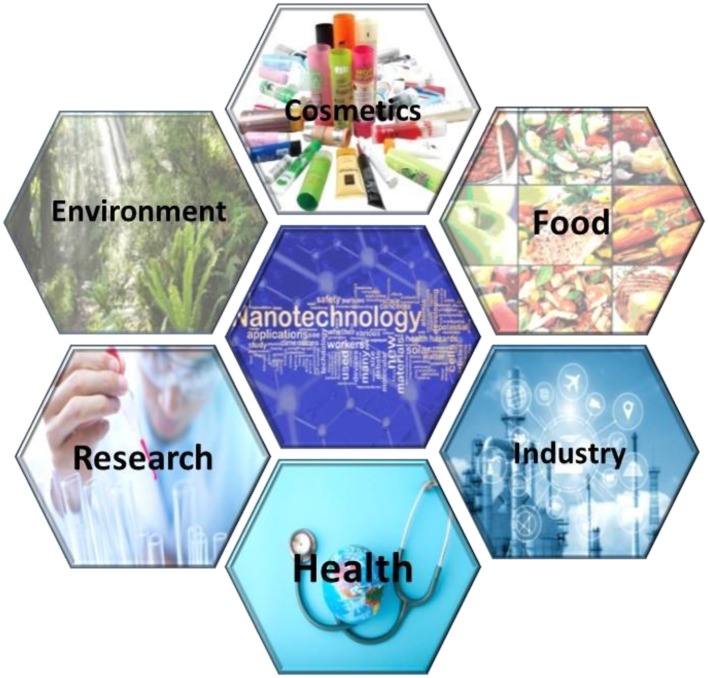
Applications of NPs in different fields.

Biomedical application is one of the top three applications of nanotechnology and it is forecasted to reach 1 trillion USD by 2024 (Inshakova and Inshakov, 2017; He et al., 2019). Tunable mechanical strength, magnetic, electrical/electrochemical, thermal and biological functionality has made them tremendous usage in healthcare, environment and energy/automobile industries (Andersson et al., 2002; Paknikar et al., 2005; Joo and Zhao, 2008). The applications of nanomaterial in different areas are illustrated in Figure 1.

Optical properties like light absorption, scattering and optical wavelength resonance made NPs more efficient in biological imaging and photo thermal therapeutics (Jain et al., 2006). NPs have shown promise in the challenging processes like detection and staging of diseases like cancer (Fortina et al., 2007; Baetke et al., 2015). Capturing the circulating tumor cells in the body of patient could be useful in targeted therapies, NPs can be used to capture these kind of cells (Miller et al., 2010). The clinical use of NPs has certain limitations due to pharmacokinetics like elimination, however they are finding important applications in understanding patho-physiological conditions of many diseases (Crich et al., 2006; Jakhmola et al., 2012). Presence of pathogens can be detected using NPs by means of immunological reactions, also the biosensors developed using NPs are useful in detection of analytes for the presence of various biomolecules like nucleic acids and proteins (Varshney and Li, 2007; Fu et al., 2014). Dozens of nanoparticle-based drugs and drug delivery systems are approved by FDA in last few decades. Bobo et al. have systematically presented the list of these drugs in their article, where one can see the applications of NPs in diseases like, multiple myeloma, pancreatic cancer, multiple sclerosis, hemophilia, hepatitis C and so on (Bobo et al., 2016). Drug delivery is another challenge in therapeutics. Drugs like anticancer agents, anti-HIV drugs, DNA strands in gene therapy have been successfully delivered using NPs, in various studies (Garcia et al., 2019).

Considering bones as complex nanocomposites, the NPs made of different metals, metaloxides, calcium phosphate, ceramic are commonly used in scaffold development for tissue regeneration (Fathi-Achachelouei et al., 2019). Besides scaffold development NPs are useful as carriers in tissue engineering to deliver drugs controllably and molecules like DNA probes, genes and growth factors. NPs with antibiotics adsorbed on the surface can be used in scaffolds to avoid bacterial infections (Wilson et al., 2010; Vieira et al., 2017). Properties like conduction and surface conjugation in gold NPs and antimicrobial properties of silver NPs or mechanical strength in carbon nanotubes have attracted researchers from tissue engineering toward application of NPs. Gold NPs are useful in promoting the proliferation rate in bone cells whereas TiO_2_ NPs are used in cell proliferation in cardiac cells (Dykman and Khlebtsov, 2012). Furthermore, NPs have shown promising intervention development of implants, especially dental implants with coatings of different NPs have shown increased stability. Nanocoatings of TiO_2_ NPs or hydroxyapatite resulted in corrosion resistance and greater integration of osseous tissues to coat implant surfaces (Yazdani et al., 2018). Other implants such as prostheses, catheters can be coated with different NPs like silver or titanium to enhance their antibacterial potential as well as stability (Ziabka et al., 2018).

### Nanotoxicity and Its Clinical Importance

**Figure 2:**
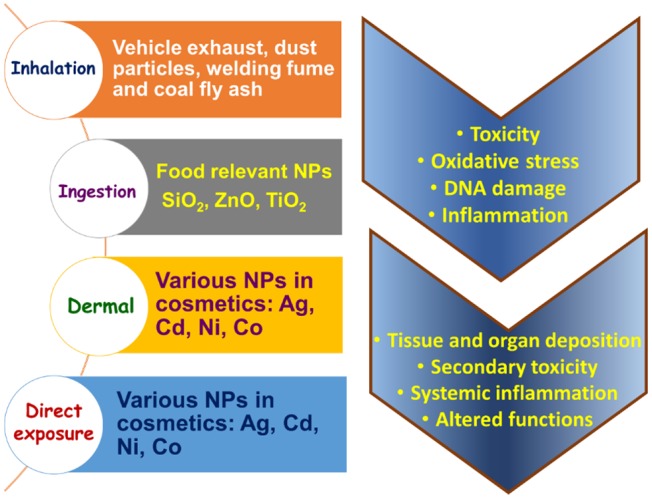
Sources and routes of NPs into the human physiology.

Major cause of nanomaterial-based toxicity is due to the unintentional release in the environment through several human activities. Especially rapid increase in use of nanotechnology in biomedical sciences and medicine such as advancements in drug delivery to specific locations in tissues, cells, different compartments and organs has raised the deliberate exposure of human to nanostructures. Nanomaterial are also responsible for many health conditions in human, like respiratory ailments, lung cancer, cardiac problems such as arrhythmia and neurodegenerative disease like Alzheimer's or Parkinson's (Buzea et al., 2007). Figure 2 represents the different sources and routes for NPs to enter the human body and their toxic effects. NPs such as ZnO, TiO_2_ are popularly used in many daily use cosmetics and food items, respectively. This has parallelly raised concerns about toxicities like; cytotoxicity, immunotoxicity, and genotoxicity due to nanocarriers itself (Blinova et al., 2010; Sharma et al., 2012). This has posed an urgent need to develop dependable procedures and techniques to characterize and assess different toxicities and hazards of the nanomaterials. These tools should be helpful to evaluate the benefits as well as the problems in use of the nanomaterials in different fields like health and environment. Although, Organization for Economic Cooperation and Development (OECD) has established a Working Party on Manufactured Nanomaterials (WPMN) in 2006 which evaluates and modifies, existing guidelines as per requirements for their applicability to test nanomaterials, the guidelines are limited to few areas only, like genotoxicity (Hougaard et al., 2015).

- *Oxidative stress and ROS generation:* Very common toxicity associated with NPs is direct or indirect generation of ROS (Table 1). The ROS generation is well-known to be involved in several health issues like aging, mutagenesis, carcinogenesis etc. It is known to induce transcription factors like NF-κb, involved in pro-inflammatory activities. The ROS generation by NPs can be attributed to their composition, size, shape, high specific surface area, concentration and high surface reactivity (Oberdörster et al., 2005; Li et al., 2008; Nel et al., 2009). The processes like release of toxic ions, intra/intercellular transport of electron or ions, lipid peroxidation also led to generation of ROS (Kamat et al., 2000; Auffan et al., 2008; Xia et al., 2008).
- *Platelet and blood:* Primarily blood is the tissue which encounters most of the NPs via different routes (Smock et al., 2014). However, no systematic studies are available to evaluate toxicities of different NPs in blood cells. There are few reports which shows silver NPs at higher concentrations results in lysis in RBCs, damage in cell membrane, hemagglutination, alterations in cytoskeletons and other morphological variations (Kim and Shin, 2014). Some of the nanomaterials such as carbon NPs are seen to increase risk of vascular thrombosis in rat model by platelet aggregation (Radomski et al., 2005). The interactions of nanomaterials with platelets may provoke inflammatory response in lungs and led to severe adverse pulmonary events (Table 1). In other interactions of platelets with nanomaterial may result into death due to multiple organ dysfunction (Chen et al., 2015).
- *Apoptosis and cell adhesion:* The toxic effects of NPs also interfere with the adhesion capacities of cells along with increased apoptosis. It may be result of overexpression of genes involved in cell death such as MA-3, p53, Bad or downregulation of genes involved in cell survival and proliferation (Alazzam et al., 2010). NPs induce apoptosis and necrosis in many cell types, in a study it is found that SiO_2_ NPs induced apoptosis in skin fibroblasts. The increased apoptosis in these cells may be the result of upregulation of pro-apoptotic genes Bim, Bax, Puma, and Noxa, along with caspase-9 (Krȩtowski et al., 2017).
- *Brain:* NPs may also cause neurotoxic effects and other secondary toxicities like disturbance with neurotransmitter metabolism by accumulating in the brain (Poli et al., 2012). In experiments to study developmental toxicities associated with NPs, it is found that the NPs can cross the placental barriers in certain cases depending upon the method of administration that is; inhalation or intravenous injection etc. The NPs may exert their toxic effects by generation of ROS, inflammatory response in mother as well as in fetus (Hougaard et al., 2015). Despite the highly regulated transportation mechanisms across blood brain barrier, the NPs may cross it by different mechanisms like transcytosis pathways such as transportermediated transcytosis, receptor-mediated transcytosis, and adsorptive-mediated transcytosis, further accumulation of NPs in the brain may be enhanced by inhibition of efflux pumps (Liu and He, 2017).
- *Hepatic and renal:* Although lungs are primary targets of nanoparticle toxicity they can reach to other organs like liver, kidney and brain through blood circulation, where they provoke immune system and increase inflammation too (Parivar et al., 2016). Being the main site for metabolism of external materials liver cells are more prone to expose to these materials which could result in to hepatic cell injury and abnormal liver function (Sidhu et al., 2004; Ahamed et al., 2013). Kidneys are another important organ for nanotoxicity as it has high blood supply and it has ability to concentrate toxin. Ina study kidneys are found susceptible to cadmium toxicity, but the NPs containing cadmium are even more dangerous as compared to its bulk counterpart (Jeng and Swanson, 2006).

**Table 1:** Toxicities of different NPs observed in various cells.

**S. no**.	**NPs**	**Cell types/lines**	**Toxicity observed**
1	SWCNTs	Human MSTO-211H cells	Toxicity was found to increase from well dispersed CNTs to
			asbestos and then to agglomerated CNTs
2	THF/nano C60	Human dermal fibroblasts, liver carcinoma cells, astrocytes	mROS, lipid peroxidation, cell death
		RAW 264.7 macrophages	ROS generation
		Human umbilical vein endothelial cells	G1 cell cycle block
		Human lens epithelial cells	Intracellular peroxides, apoptosis (phototoxicity)
3	CdSe/ ZnSSSA	EL-4 cells	Cytotoxic
		Vero, HeLa, and primary human hepatocytes	Cytotoxic, hepatocytes
4	Cu	Two humans pulmonary cell lines (A549 and THP-1)	Cytotoxic, pulmonary inflammation, collagen accumulation
5	ZnO	BEAS-2B and RAW 264.7 macrophages	Increased intracellular calcium, decreased mitochondrial membrane potential, interleukin 8 productin
		Neuro-2A cell line	
6	Ag	Murine macrophage cell line	Cytotoxic
		Mouse spermatogonial stem cells	Mitochondrial damage, enhanced formation of autosomes and autolysosomes, downregulation tight junctions
7	(CeO_2_)	Human lung carcinoma (A549)	Cytotoxic through ROS generation and reduced glutathione
8	Au/CeO_2_	Human hepatocyte (C3A), human colon adenocarcinoma (CaCo-2), primary trout hepatocytes	Cytotoxicity effects
9	SiO_2_	Mouse spleen	Decreased proliferation in B-cell and T-cell, decreased interleukins (IL-12. IL-6)

## State of the Art Model Assay Systems for Nanotoxicology

**Figure 3:**
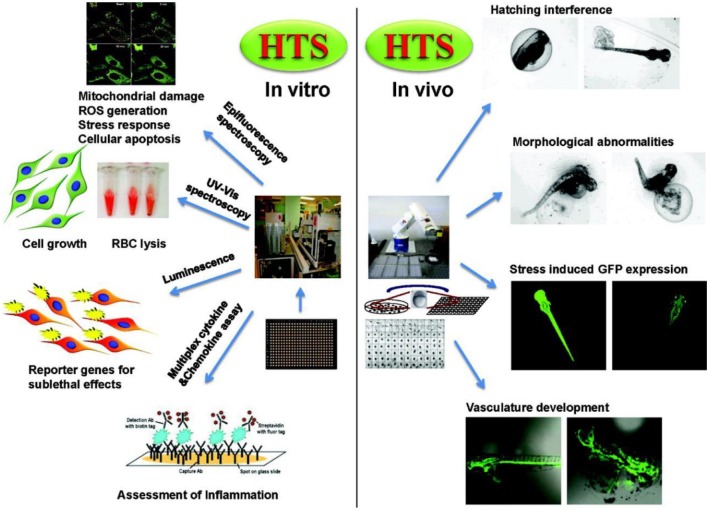
Examples for *in vitro* and *in vivo* high throughput assays and response readouts in cells and zebrafish embryos. Reproduced with permission from Nel et al. (2012).

The second infrastructure requirement for a predictive toxicological approach is the development of appropriate high-throughput screening approaches to quantitatively assess dose- and time-dependent cellular injury responses that are predictive of *in vivo* adverse outcomes (Figure 3). Biological, medical, pharmaceutical, and toxicological research has illustrated how a systems biology approach can be used for high-throughput screening. Although the current approach to the risk assessment of toxic substances (such as chemicals) is heavily reliant on apical adverse health effects in animal models, a description of adverse health outcomes does not provide a robust science-based platform for performance of predictive toxicological modeling (Nel et al., 2012).

**Figure 4:**
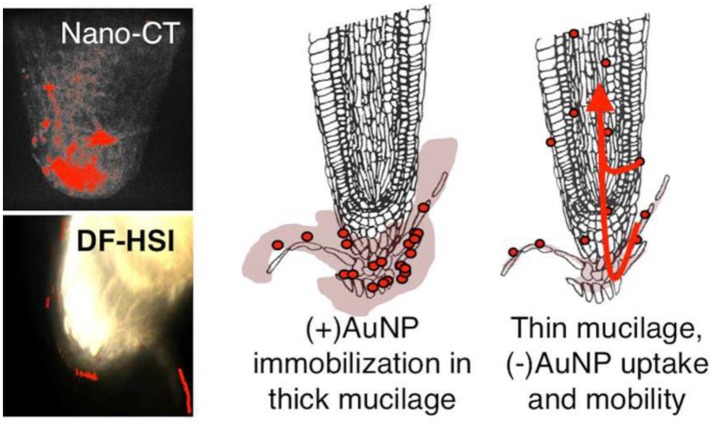
Direct evidence for charge specific translocation of AuNPs (+ve and ve charged) in root of *A. thaliana*. Adapted with permission from Avellan et al. (2017), *Environ. Sci. Technol*. Copyright 2017, ACS Publications.

Scientific evidence on physiological effects of bio-functional nanomaterials are highly desired for both acute and chronic applications. In this context, bioassay models derived from both the plant and animal species for assaying the toxicity of novel materials, particularly the nanostructures and their impact on biological systems have elaborated the toxicity profile usefulness for optimizing their health care utilities. Among the wide varieties of plant species, *Arabidopsis thaliana* have been established as an elegant model system owing to its rapid life cycle (6 weeks from germination to seed maturation), simple cultivation in small space and well-revealed genomic information (~114.5 Mb). Recently several nanotoxicity studies have been attempted involving the model, *A. thaliana*. For instance, Kaveh et al. (2013) demonstrated the gene expression investigation on *A. thaliana* upon interaction with pristine Ag^+^ and AgNPs. Experimental results suggest that *A. thaliana* has exposed an up-regulation of 286 genes and down-regulated genes of 81 due to AgNPs, which is different from interaction with pristine Ag^+^. During the seedlings period, *A. thaliana* were exposed to 20 mg L^1^ of AgNPs (average size: 20 nm) or AgNO_3_ for a period of 10 days, afterward the leaves and roots were harvested for genomic analysis using microarray technique. Mechanism behind the up-regulation and down-regulation genes were correlated with the metal associated oxidative stress and pathogens, in addition to hormonal stimuli. Further, the AgNPs induced salt stress, insecticide/anti-infective property, wound healing and thalianol biosynthetic pathway were also correlated for up-regulation of genes. Another study by Nair and Chung (2014) demonstrated that AgNPs interaction with *A. thaliana* has resulted in acceleration. With advent of modern imaging tools, researchers have attempted to map the internalization and translocation of optically active NPs in the plants. For instance, Avellan et al. (2017) have used two complementary imaging techniques based on X-ray nanotomography and enhanced dark-field microscopy integrated with hyperspectral imaging to visualize the interaction of positively and negatively charged Au NPs with roots of *A. thaliana* (Figure 4). These observations not only provide direct insight on the NPs-plant interactions but also provide feasibility for environmental analysis of toxicants in the terrestrial species (Avellan et al., 2017).

**Figure 5:**
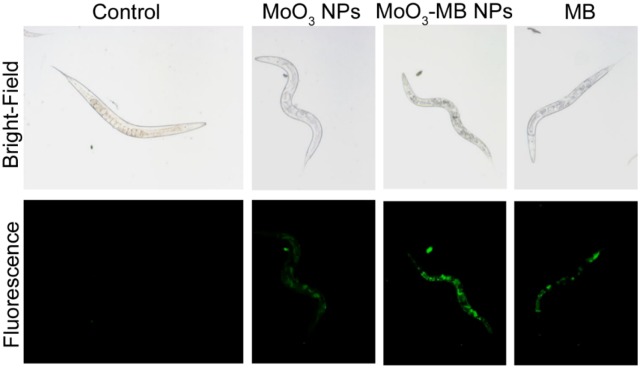
Fluorescence microscopic images of *C. elegans* after exposure (12 h) with 10 μg mL^−1^ of NPs of MoO_3_, MoO_3_-MB NPs and pristine MB. Adapted with permission from Marimuthu et al. (2018), ACS Applied Mater. Interfaces Copyright 2018, ACS Publications.

Similar to plant species, animal models-based bioassay, apart from conventional mouse/rat, using worm, *Drosophila* and zebrafish were widely explored for elucidation of nanotoxicology and nanotherapeutics. For instance, Kim et al. (2017) studied the cellular uptake and toxicity assay of AgNPs against *Caenorhabditis elegans* using a customized microfluidic chip measuring the worm length/width, behavior (movement) and level of metallothionein (*mtl*-2) gene expression. Among the studied interactions, of AgNPs, Ag^+^ and Cd^2+^, only the pristine AgNPs expressed both the *mtl*-2 gene expression and growth inhibition. This correlation was assigned due to the colloidal metallic nature and nanoparticular structure of Ag. Yang et al. (2017) assessed the bioaccumulation and nanotoxicity of zerovalent Fe NPs against *C. elegans* proposing a toxicokinetic and toxicodynamic modality implicating the fertility of *C. elegans*. Apart from toxicity studies, therapeutic application of novel nanomaterial was also recently explored in *C. elegans*. For instance, Marimuthu et al. (2018) have demonstrated an intracellular bioimaging and *in vivo* anti-oxidant property of molybdenum trioxide (MoO_3_) and methylene blue-modified molybdenum trioxide NPs (MoO_3_-MB NPs) using *C. elegans* bioassay model. Figure 5 illustrates the fluorescence microscopic images of *C. elegans* before and after interaction with derivatives of MoO_3_ NPs. The stress-associated markers and metabolic pathways of *C. elegans* are closely related to human biochemical pathways, thus studying their physiological response against acute or chronic antiinflammatory and neurological therapeutics are useful. Radical scavenging materials are highly beneficial for cancer and anti-inflammatory therapeutics. To evaluate the anti-oxidant behavior of MoO_3_-MB NPs, a controlled heat shock was given to *C. elegans* which in turn created an oxidative stress within the cellular system elevating intracellular ROS and hydrogen peroxide (H_2_O_2_).

Upon treatment with hybrid MoO_3_-MB NPs, *C. elegans* were able to heal their oxidative stress, which are evidenced from the expression level of stress rescue markers such as glutathione peroxidase and catalase. Similar to standard antioxidant resveratrol, the ROS reduction capability of MoO_3_-MB NPs and MB against H_2_O_2_ was better than the bare MoO_3._ Lethal and sub-lethal dose for MoO_3_-MB NPs against *C. elegans* was found to be 40 μg mL^1^ and 10 μg mL^1^. Similarly, *Drosophila melanogaster* has gained significant popularity in nanotoxicity assay. For instance, Ong et al. (2015) reviewed the biology of *D. melanogaster* with respect to different modes of nanomaterials toxicity including oxidative stress, genotoxicity, fecundity and metabolic defects. Other important species included in aquatic toxicology is zebrafish (*Danio rerio*) which is effective for testing small molecules and moreover exhibit physiological response comparable with mammals, having anatomical coherence such as blood brain barrier, endothelial cells and immunogenic response. For instance, in the event of interaction with xenobiotics it can produce biochemicals that can fight against oxidative stress and foreign bodies. Thus, it can also offer a better model for studying the respective nanomaterials which tend to be used in drug delivery for targeted therapeutics. In this aspect, de Souza Filho et al. (2014) studied the *in vivo* genotoxic effects of multiwalled CNTs with an average diameter of 500 nm (de Souza Filho et al., 2014).

## Electrochemical Assessment of Nanomaterials for *in vitro* Healthcare

Correlating the cellular toxicity and nanostructure bio-functionality have often been studied by exploring the inherent changes occurring in the intracellular system through monitoring the cellular events. This includes the measurement oxido-reductase enzymes *viz.*, nicotinamide adenine dinucleotide, dehydrogenase and neurotransmitters such as dopamine, ROS such as hydrogen peroxide (H_2_O_2_) and superoxide dismutases (SOD) and reactive nitrogen species (RNS) like nitric oxide (NO), peroxynitrite (ONOO-) and nitrogen dioxide (NO_2_) (Zhou et al., 2009). The most commonly used analytical methods for cell toxicity analysis are microplate readers, flow cytometers and high content imaging. Most of the cell viability methods measure metabolites produced or enzymatic activity as markers of viable cells. Viability methodologies based on enzymatic activity usually involves the use of optical detection protocols such as absorbance, fluorescence and luminescence. The enzymes involved in such protocols are protease, oxido-reducatases, and esterases. Analytical electrochemistry is becoming an important tool for assessing the neurological and biophysiological processes in cells upon their interaction with nanomaterials. Electrochemical methods can be used for the high throughput analysis of electroactive molecules present in the cell population. Non-electrochemical molecules can also be detected by electrochemical methods by designing suitable biorecognition probes. Electrochemical and bioelectrochemical methods are becoming important tool in analyzing and screening the toxicological effects of engineered nanomaterials. There are several areas in which the electrochemistry can contribute to the nanotoxicity assessment studies, or as complementary or alternative analytical tool to the conventional cytotoxicity analysis methods. EIS can probe the frequency dependent electrical impedance of cells upon interaction with nanomaterial (Zhang et al., 2012). For example, Hondroulis et al. have designed an electrical impedance measurement array on chip for quantifying the toxicological effects of NPs such as silver, gold, and cadmium oxide and single-walled carbon nanotubes on human lung fibroblasts and rainbow trout gill epithelial cells (Hondroulis et al., 2010). Electrochemical methods coupled with enzymatic assay has been investigated to measure the cell viability. The concept is based on measuring the changes in cellular enzyme activity (acid phosphatase) upon exposure with the nanomaterials. The enzymes are located in cell membranes, cytosol, and in lysozymes. Mammalian lung epithelial A459 cells were used as a model cell line and cadmium telluride quantum dots (CdTe QDs) was selected as a model nanomaterial toxin for the electronic assay (O'Hara et al., 2017). The cells were immobilized in a 96 well-plate and exposed to CdTe QDs. The acid phosphatase catalyses the conversion of the organic phosphate molecule, 2naphthyl phosphate to 2-naphthol. The cellular generation of redox active enzymatic product.

2-naphthol was detected at a modified screen-printed electrode surface using cyclic voltammetry and chronocoloumetry techniques. Quantitative detection of extracellular H_2_O_2_ released from the cells upon exposure to nanomaterials have also been studied using electrochemical techniques.

Different types of mechanisms including cellular exocytosis, production of reactive oxygen species (ROS)/reactive nitrogen species (RNS), measuring the impedance behavior of the cells, and monitoring ions released have been reported for studying the cytotoxic effect of nanomaterials. The attractive characteristics of electrochemical methods are becoming important in studying the different mechanistic aspects of nanomaterial toxicity. The electrochemical tools that are employed for monitoring cellular toxicity is categorized into different types as discussed below.

**Figure 6:**
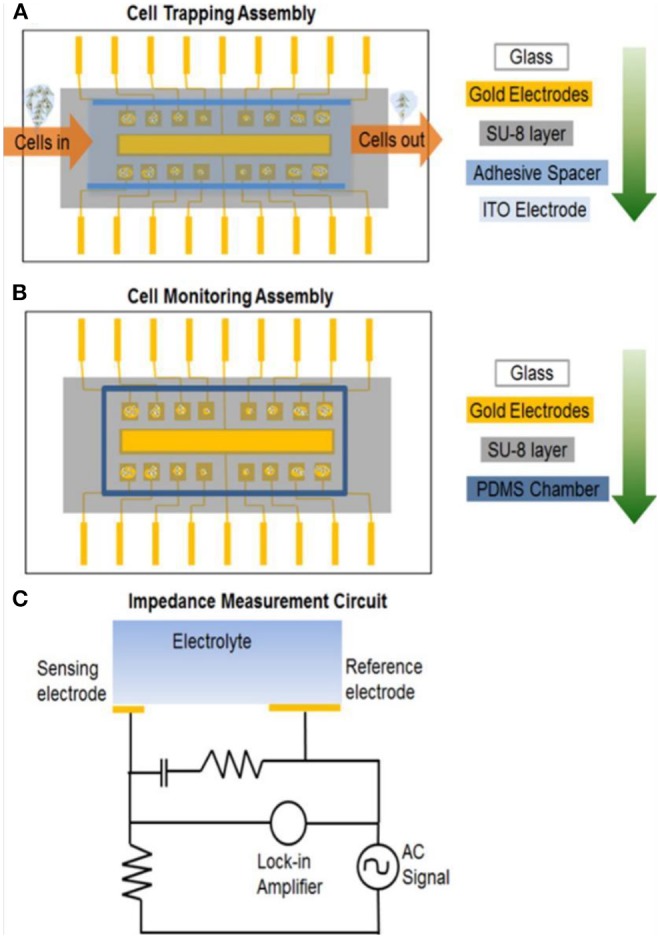
Nanotoxicity analysis on chip. **(A)** MEMS device assembly for dielectrophoresis trapping of cells; glass wafer is the base, gold sensing electrodes are under the SU-8 microwell pattern, spacer is user to hold the top ITO electrode. **(B)** MEMS device assembly when top ITO electrode is removed and PDMS chamber is placed to contain cell growth solution while monitoring cell impedance. **(C)** Electronic cell impedance sensing circuit representing a single sensing electrode for simplicity. Reprinted with permission from Shah et al. (2016).

**Figure 7:**
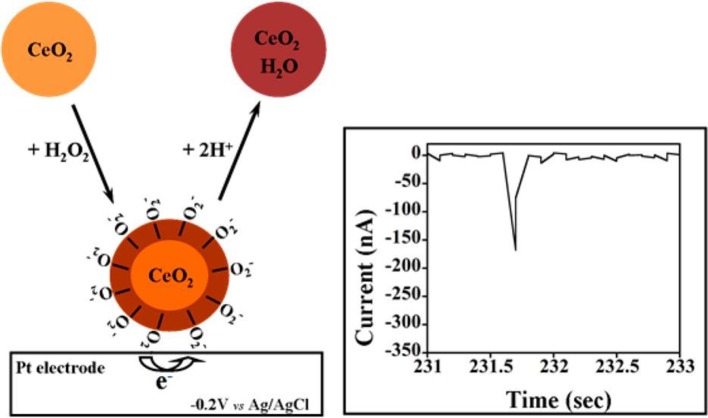
Schematic Illustration of Single CeO_2_ NP Collision Events and the Reduction Current Spike for the Ce–O_2_–/O_2_^2^– NPs in Contact with The Pt ME (φ = 125 μm). Reprinted with permission from Sardesai et al. (2013).

- *Microelectrodes performing amperometric measurements for monitoring cell exocytosis and neurotoxicity events in systems exposed to nanomaterials*.
- *Electrochemical evaluation of cellular oxidative stress response to investigate the changes in ROS/RNS profile in real-time*.
- *Utilizing ECIS for monitoring the cellular biophysical changes upon interaction with the nanomaterials*.
- Potentiometric analysis for measuring the ions released in the cellular systems and
- *Use of electrochemical-collision methods to screen the particle reactivity, surface coating, and physicochemical properties*.

- *Amperometric microelectrodes for assessing the cellular cytotoxicity:* Carbon fiber microelectrodes performing amperometric measurements have been used to monitor the dynamic section of chemical messenger molecules from cells exposed with nanomaterials. This method can allow the quantitative detection of specific messenger molecule based on applied potential at submillimolar time resolution scale. Unlike conventional glassy carbon electrode or screen-printed electrodes, custom-designed microelectrodes possess typical advantages particularly in probing the biochemical changes at small groups of cell culture. Carbon-fiber microelectrode amperometry (CFMA) has been demonstrated to monitor the serotonin exocytosis from murine peritoneal mast cells (Marquis et al., 2008). Exocytosis is an organized cell function across cell types and plays crucial roles in chemical communication between the cells. With the help of CFMA, researcher can monitor the cell exocytosis process and correlate changes in single-cell exocytosis to bulk measurements such as NP uptake and cytotoxicity. The amperometric measurement can be beneficial in providing the biophysical mechanistic information on how the nanoparticle is influencing the cell behavior. Haynes's research group demonstrated carbon fiber amperometry to evaluate the influence of NPs on the release of serotonin during exocytosis in cells. The study explored the effect of three different metal oxide NPs (non-porous SiO_2_, mesoporous SiO_2_, and TiO_2_) on the toxicity of mast cells, a type of cell line known for its exocytotic delivery of chemical messengers (Maurer-Jones et al., 2010). These NPs are widely used as additives in commercially available products, and play important role in biomedical research. Nanocomposites of these materials finds critical applications in therapeutics, diagnosis, imaging, and drug delivery. Effect of particle size and crystallinity of the materials on the cytotoxicity has also been investigated. The research group also investigated the effect of gold NPs on the cellular cytotoxicity. They have used CFMA to study the serotonin exocytosis from murine cells upon interaction with gold NPs (Marquis et al., 2008). Similarly, Andreesscu team investigated the effect of ceria NPs
  (CeO_2_) on serotonin exocytosis in live zebrafish model (Özel et al., 2013b). Nanoceria or CeO_2_ NPs is known for its potential role as therapeutic agent in the treatment of oxidative stress and related diseases. Typically, the intestinal serotonin under the impact of CuO, Ni and CeO_2_ NPs were assessed using an implantable CFME. It was substantiated that the alternations in the intestinal serotonin levels might be due to the organ dysfunction caused by NPs exposure in the embryos. Further the experimental observation suggests that the DPV results were in coherence with immunohistochemistry of the dissected intestine, enabling the possibility of electrochemical strategy for nanotoxicity analysis. The CeO_2_ NPs are reported to have neuroprotective effect and known to cross blood brain barriers (Estevez et al., 2011). The group also investigated the impact of two different NPs, Ni and CuO on the release of serotonin from the intestine of zebrafish embryo using implanted CFME (Özel et al., 2014). Similarly, Njagi et al. used implantable CFMEs for monitoring the physiological expression of serotonin within the zebrafish embryos intestine at high spatial resolution and specificity using differential pulse voltammetry (DPV) measurements. The microsensor can detect the pharmacological elevations in serotonin release and provide the longitudinal distribution of the neurotransmitter level along the intestine with high spatial resolution (Njagi et al., 2010).
- *Electrochemical tools for assessing cellular oxidative stress response:* Another important mechanism for nanomaterial induced cytotoxicity is the overproduction of ROS and RNS, in cells resulting in the subsequent formation of oxidative stress to the tissues (Qiu et al., 2018). To understand the mechanism at the molecular levels, the interaction of engineered nanomaterials (ENMs) with the living systems has been studied. The ROS induced toxicity leads to DNA damage, changes in cell motility, and unregulated cell signaling. The ROS production and its cytotoxic effects of quantum dots have been reviewed by Winnik and Maysinger (Winnik and Maysinger, 2012). The acute cytotoxicity and oxidative stress effect of metal oxide NPs such as TiO_2_ and ZnO has been studied in zebrafish model. Wang et al. (2012) have developed electrodes comprising nanostructures of Pt/Pt black for voltammetric sensing of intracellular ROS and RNS in macrophages. The production of ·OH radicals as result of nanomaterial interaction with cells is assayed using five different oxidative stress biomarkers (Xiong et al., 2011). Electrochemical methods have been demonstrated to monitor the production of ROS/RNS in cells and their subsequent DNA damage. Gasper reviewed the electrochemical sensors used for monitoring the production of superoxide and hydrogen peroxide in living cells (Gáspár, 2011). Role of CeO_2_ NPs on the release of RNS in zebrafish model was also investigated (Özel et al., 2013a). They have studied the impact of metal oxide NPs such as CuO and CeO_2_ in altering the intestinal NO concentrations changes in zebrafish. NO selective carbon microelectrodes were used to measure the NO release. The study revealed that CuO NPs at higher concentrations induced the intestinal oxidative damage and released a greater number of NO when compared to CeO_2_ NPs, which acting as a NO scavenger. Similarly, the cytotoxic effect of different metal oxide NPs such as CuO, TiO_2_, ZnO, CuZnFe_2_O_4_, Fe_3_O_4_, Fe_2_O_3_ were investigated and compared with the carbon NPs and MWCNTs (Karlsson et al., 2008). DNA damage, oxidative stress and production of intracellular ROS were measured to correlate the impact of the nanomaterials. The intracellular production of small molecules including lactate or glucose can be detected using lactate oxidase or glucose oxidase immobilized electrodes, while the ROS/RNS generation can be detected by using cytochrome c (cyt c) integrated electrodes. For an example, gold electrode integrated with cyt *c* was used to detect the effect of CeO_2_ NPs on ischemic brain slices (Ganesana et al., 2012). The immobilized cyt c is reduced by superoxide and the reduced cyt *c* is regenerated electrochemically at the electrode surface. The concentration of superoxide was quantified by constant potential amperometry with the electrode poised at the potential of 0.15 V vs. Ag/AgCl, which corresponds to the oxidation potential of Cyt c. The exposure to CeO_2_ NPs induced an immediate decrease in the superoxide signal under control conditions. In addition to ROS/RNS and neurotransmitters, the other classes of important target molecules for nanotoxicity analysis are glucose, sialic acid, C-reactive protein (CRP), lactate and glutamate. This is because of their expression level at acute infectious stage, which is regarded as critical for diagnosis of fatal disease. For improved sensitivity and stability of electrochemical sensor platform, researches have been attempted with variety of chemically modified electrodes especially using carbon-based nanostructures, due to its cost-efficient scalable preparation. For instance, metalloid NPs (Ag@SiO_2_) modified with polymer (polyethylene glycol) and 2D carbon nanostructure like graphene oxide (GO) offers enhanced stability and selectivity toward both the serum and urine glucose (Veerapandian et al., 2012). Amino sugar glucosamine modified GO nanosheets upon UV irradiation exhibited improved electron transfer ability at the electrode-electrolyte interface. UV-irradiation on the glucosamine-modified GO nanosheet inherently alters the composition of carbon-oxygen and nitrogen elemental composition potential for enzymatic electrocatalysis of target sialic acid (Veerapandian et al., 2015). In an earlier study, glucosamine-modified copper microstructure studied to have compatible environment for immobilization of monoclonal antibodies specific to CRP antigens (Veerapandian et al., 2011).
- *Utilizing ECIS for monitoring the cellular biophysical changes upon interaction with the nanomaterials:* Similar to electrochemical nanotoxicity, recently electrical resistance-based cytotoxicity analysis, including cell attachment and spreading, differentiation, proliferation, invasion, inflammation and barrier function, is emerging for various pharmaceutical and molecular biological application. Electrochemical impedance spectroscopy (EIS) combined with microelectromechanical systems (MEMS) is becoming important tool in studying the nanotoxicity assessment of single cell and small cell populations. Cells and tissues can adhere to the electrodes, and could change the cell-electrode interfacial properties, which will result in the alteration of electrode impedance and capacitance. Thus, EIS can be used to probe the alterations in morphology and density of cells, cell viability, and extent of cell attachment. Measuring cell impedance have shown to provide several advantages for nanotoxicology assay compared to the conventional cytotoxicity screening methods: (i) the EIS measurement are not influenced by absorptive properties or chemical activity of NPs; (ii) the optical properties of the NPs are not a limiting factor in toxicity assay as the quantification in EIS is based on the cell morphology; (iii) the EIS system could provide online measurement and continuous sampling, providing valuable information on the reaction rates and mechanisms of cellular events. The inherent toxicity of nanomaterial interrupts the cell behavior and changes the cell impedance which can be monitored in real-time using EIS. Number of electrochemical techniques have been reported to access the cell viability following the exposure to toxic nanomaterials. Electrical cell-substrate impedance sensing system (ECIS) is a label-free non-invasive bioassay technique equipped with microelectrode array capable of acquiring the structural and functional properties of living cells as the function of resistance in real time. ECIS to monitor mammalian fibroblast cells was initiated by Giaever and Keese (1984). Mammalian fibroblasts have been cultured on evaporated gold electrodes subjected to an alternating electric field at 4000 Hz. Breus et al. have studied the cytotoxicity behavior of the fluorescent rich negative charged QDs, CdSe/ZnS core/shell, in comparison with positively charged and zwitterionic QDs exposed to MDCKII kidney cells using ECIS system (Breus et al., 2015).
  The toxicity of graphene nanomaterials was investigated against the *in-vitro* blood brain barrier (BBB) model by measuring the trans-endothelial-electrical resistance (TEER) using EIS chip (Hondroulis et al., 2012). Graphene showed no significant toxicity against the BBB model and cellular components, suggesting its potential applications as vehicle for drug delivery. Similarly, the impact of metal NPs (Au and Ag), SWCNTs, and CdO on cytotoxicity were compared (Hondroulis et al., 2010). EIS was used to track the nanotoxicity over time and provide insights into the early and pronounced dose dependent toxicity. The time dependent cytotoxic effects of commercially available metal oxide NPs such as CuO, ZnO, and TiO_2_ toward lung epithelial cell lines are compared using EIS (Seiffert et al., 2011). EIS integrated with real-time cell analyser (RTCA) can provide high throughput toxicity screening. RTCA involves interdigitated electrodes attached to the bottom of 96-well cell culture plates for high throughput screening. Using the RTCA system, the cytotoxicity effect of 11-different inorganic nanomaterial against human bronchial epithelial cells were analyzed (Otero-González et al., 2012). Pratikkumar et al. studied the cytotoxic effects of NPs on a single cell as well as on small cell populations using EIS and a microelectromechanical system (MEMS) device (Shah et al., 2016). The designs used for cell-trapping and cell-monitoring is depicted in Figure 6. ITO electrodes were used to generate the required waveform and double-sided adhesive tape is used to create microfluidic channels. The impedance-based detection systems are becoming useful in analyzing wound healing mechanisms. For instance, Gamal et al. have developed an age-related macular degeneration (AMD) disease model-on-a-chip using human induced pluripotent stem cells cultured on ECIS system for studying the wounding assay (Breus et al., 2015). An electrical model assay for investigating wound-healing capability of keratinocyte cells under different pressures using wound closure speed, cell power and cell electroactivity (Yang et al., 2016). Similarly, Cavallini and Tarantola have studied the wound closure dynamics of mono- and co-cultures of myocytes and fibroblasts under ECIS for understanding the crosstalk of major heart cell groups (Cavallini and Tarantola, 2019).
- *Potentiometric analysis for measuring the ions released in the cellular systems:* Potentiometric biosensors thus have been developed for cytotoxicity applications for measuring the electrochemical potential change (Koncki, 2007). This type of electrical measurement is useful in studying the behavior of cells in relation to the membrane potential of nerve and muscle cells and monitoring the adhesion of cells (Woolley et al., 2002). Cells which are attached to the electrode surface will form a cell-sensor interface. The electrochemical signal at the cell-sensor interface can be used to probe the kinetics and dynamics of the transport across the cell membrane. Open circuit potential measurements were performed to investigate the cytotoxic effects of surface metal oxides against lung epithelial cells (Hedberg et al., 2016). The potentiometric ion-selective electrodes are becoming important tool in monitoring DNA damage (Numnuam et al., 2008) caused by nanoparticle induced cytotoxicity. Potentiometric sensor arrays for investigating the cytotoxicity with potential application is drug discovery has been demonstrated (Wang et al., 2010). A filed effect transistor (FET) based biosensor for ATP has been developed using nanopipette modified pyrolytic carbon nanoelectrodes (Zhang et al., 2016). The source and drain were coated with electrodeposited polypyrrole (PPy), which forms a transistor channel.
  Hexokinase enzyme was immobilized on the FET electrodes for catalyzing the electrochemical conversion of adenosine triphosphate (ATP) to adenosine diphosphate (ADP) and to cause the phosphorylation of glucose and subsequent release of a proton. This used to record ATP concentrations near and inside living cells.
- *Electrochemical-collision methods to screen the particle reactivity:* Electrochemical interaction and collision of the nanomaterials at the electrode surface can be used to assess the catalytic as well as physiochemical properties of the nanomaterials. The nanomaterialelectrode collision characteristics depends on the electrode surface coting, structure-activity relationship, and collision time duration. The collision profile of platinum NPs with an ultramicroelectrode were observed electrochemically by monitoring the current-time transients for a particle-catalyzed reaction (Xiao and Bard, 2007). Compton et al., investigated the particle coulometry to investigate the collision profile of tagged silver NPs with carbon electrodes. This method can be useful in identify the nanomaterials labels used in microfluidics and analytical science (Zhou et al., 2012). Similarly, our group recently studied the cytotoxicity of chitosan-gold nanocubes nanocomposites used in the cytochrome *c* sensor construction (Manickam et al., 2020). The cytotoxic effects were analyzed in two different cell lines. Andrescu team developed a cost-effective electrochemical screening strategy to assess the antioxidant activity of CeO_2_ NPs by single nanoparticle collision at microelectrodes (Sardesai et al., 2013). This method can be applied to screen particles for their ability to inactivate ROS and assist with prior selection of CeO_2_ NPs candidates before more extensive cell and animal experimentation is being performed (Figure 7).

## Conclusion and Outlook

The present review compiles the recent research reports in the field of nanomaterials-derived toxicological studies. To understand the effects of nanomaterials on human healthcare, different biological models have been proposed. Still detailed characteristics of physicochemical properties including size, shape, composition, surface characteristics, and stability in different environment (culture medium) need to be studied and correlated for acute/chronic toxicity, which can eventually provide better understanding on the biological effects of the nanomaterials. Unlike classical cell biological analysis, electrochemical and electrical method of macromolecular study could elevate the diagnosis and therapeutics to the next level, supporting the bed-side tests. Modern research in biomedical science offers embedded electronics and advanced signal processing techniques useful for real-time measurement of clinical abnormalities and treatment. To achieve such unambiguous targets establishment of advanced functional nanomaterials without undesired toxicity is highly needed. Despite the stability issues in bioreceptors and batch variations in large scale manufacturing and fabrication expensiveness, still screen-printed electrode and MEMS technology based analytical devices are valuable for healthcare applications. Thus, it provides lot of opportunities for multiplexed analysis. Further, much efforts in optimizing the electrochemical cell design capable of accommodating cell lines of mono or coculture, spheroids, artificial tissues, and microbial colonies with suitable resistance against biocorrosion are desired. Research on cost-efficient electrode arrays capable of evaluating cell-cell interaction, cell-drug interaction and cell-cytotoxicity assay with sensitivity and reliability is highly demanded. Research integration involving metabolomics and proteomics study on nanomaterial-cell toxicity can also pave way for new avenues in modern healthcare. Nevertheless, the combined efforts from complete nano-bio-interface analysis, highthroughput multiscale bio-evaluation, detailed computational simulations, and artificial intelligence could help in optimizing the nanomaterial formulations for safe, efficient usage in the consumer products and clinical applications.

## Author Contributions

PM prepared the original designs and developed the idea. RS and MV articulated the manuscript with equal contribution. AK assisted in the execution of the idea and manuscript writing.

## Conflict of Interest

The authors declare that the research was conducted in the absence of any commercial or financial relationships that could be construed as a potential conflict of interest.
